# A case of misdiagnosis and mismanagement in forensic psychiatry

**DOI:** 10.1192/j.eurpsy.2024.110

**Published:** 2024-08-27

**Authors:** F. Saeedzadeh Sardahaee

**Affiliations:** ^1^Psychiatric clinic, Lukasstiftelsen; ^2^Psychiatric clinic, Advansmedical, Trondheim, Norway

## Abstract

**Abstract:**

A case of misdiagnosis and mismanagement in forensic psychiatry Speaker: Farzaneh Saeedzadeh Sardahaee, MD, PhD, Consultant Psychiatrist, Trondheim, Norway

Timely diagnosis and treatment for psychiatric illnesses in first time offenders in forensic psychiatry remains a challenge. Presenting a young male first-time offender with recent onset psychosis, ADHD and anxiety disorder, the speaker reflects upon the complexities around making the right diagnosis in a prison with very limited resources for clinical observation. Minting the term “Psychosis Incognito”, the speaker goes on to explore a complex multiagency follow up process faced by this offender and his treating team, as well as many medical complications including malnutrition and Korsakoff syndrome.

**Disclosure of Interest:**

None Declared

